# Supplementary material for: Dislodgement resistance and structural changes of tricalcium silicate-based cements after exposure to different chelating agents
Source: PLoS One. 2024 Jan 17;19(1):e0296647. doi: 10.1371/journal.pone.0296647 (PMC10793889; doi:10.1371/journal.pone.0296647)
Supplement: Attachment — Submitted filename: Review PLOSone 2.docx [file pone.0296647.s002.docx]

**Comments to the authors:**

Thank you for choosing a very interesting topic; I believe that the study is somewhat underpowered, however, the manuscript is adequately written otherwise.

Attached below are the comments for your reference:

**Abstract:**

Please use the full form of MTA and HEDP instead of the abbreviation when used for the first time in the manuscript.

**Introduction:**

- I feel that the narrative is not strong enough; the introduction is quite vague and there is some room for improvement.
- No clear description as to how the proposed alternatives have overcome the disadvantages of the conventional MTA regarding the context of the study. Do these materials exhibit better sealing ability than MTA or do they just differ in composition? Is there any existing literature regarding the effect of different irrigants on the structural properties of MTA versus the proposed alternatives?
- With reference to the proposed alternatives, is there any contrasting evidence regarding the dislodgement resistance and structural changes after exposure to different irrigants or the idea is completely novel? If this is the case, please include this in the introduction section.
- Third paragraph in the introduction is very generic and can be improved within the context of the title of the study.

**Materials & Methods:**

- No clear description as to how the sample size was calculated for the current study; the quoted reference had a single group (Biodentine) and the only objective was to determine the pushout bond strength of Biodentine when exposed to different irrigation protocols. On the contrary, the present study had three groups, and the objective was twofold i.e., dislodgement resistance and structural changes of cements were evaluated. Hence, I believe that the study is somewhat underpowered.
- Fairly adequate description of the rest of the methodology otherwise; someone who would like to perform a similar study can easily replicate it.
- Line 132: 0.2% sodium azide; is this a recommended storage medium?
- Lines 192-193: why only fifteen specimens were evaluated for microstructure analysis? Is it because it was their secondary objective; if this is the case where did this number come from as this is not mentioned in the sample size calculation either?

**Results:**

- Was the data non-normal? I am assuming this was the case as non-parametric tests were applied.
- The authors could have excluded the outliers before applying statistical analysis as this would have resulted in a normal distribution of the data but considering the sample size per group was too small, I assume this was not possible.
- Which statistical test was applied for the data in table 2?

**Discussion:**

- Discussion section is adequately written with a clear description of the findings of the present study.
