# Supplementary material for: Dislodgement resistance and structural changes of tricalcium silicate-based cements after exposure to different chelating agents
Source: PLoS One. 2024 Jan 17;19(1):e0296647. doi: 10.1371/journal.pone.0296647 (PMC10793889; doi:10.1371/journal.pone.0296647)
Supplement: Attachment — Submitted filename: Response to Reviewers_PLOSOne.doc [file pone.0296647.s003.doc]

**Responses to reviewers’ comments:**

The authors are thankful for the comments and suggestions made by the reviewers. In light of those suggestions the manuscript has been now modified. The clarifications for the comments and the modifications suggested by the reviewers are incorporated in the revised manuscript as given below.

**Reviewer #1**: Thank you very much for a very nice manuscript. I congratulate the authors on their hard work. I have very few comments as follows

**Introduction:**
Question 1: Line 68 ProRoot MTA and OrthoMTA are old products. They you have written it, it seems they are new. Please rephrase.

Our response: The word “recent” has been removed in the revised manuscript.

Question 2: Line 94: Correct HEBP to HEDP

Our response: The abbreviation “HEBP” is corrected to “HEDP”.


**Results:**
Question 1: Table legends are not adequate. Please indicate what does the two and three Asterix imply. Also what test was used should be indicated.

Our response: Titles of table 1 and 2 has been modified in the revised manuscript.

Discussion:
The Combination product NaOCl/HEDP may not be as effective as EDTA at smear removal. You need to explore this more in your discussion, since any benefit that one may get from HEDP in terms of reduced detrimental effects on MTA are negated by the lack of or poor primary function i.e. the smear removal.

Our response: The inferior ability of NaOCl/HEDP combination in removal of smear layer when compared to 17% EDTA has been added in the revised manuscript.

**Reviewer #**2: Except that the study was underpowered, all other technical aspects were taken care of and the statistical analysis was adequately performed. The introduction section is quite generic but the rest of the manuscript is adequately written otherwise and the methodology is quite crisp and easy to replicate.

**Abstract:**

Question 1: Please use the full form of MTA and HEDP instead of the abbreviation when used for the first time in the manuscript.

Our response: Full form of abbreviations MTA and HEDP has been added in the abstract of revised manuscript.

**Introduction:**

Question 1: I feel that the narrative is not strong enough; the introduction is quite vague and there is some room for improvement.

Our response: The introduction has been modified in the revised manuscript.

Question 2: No clear description as to how the proposed alternatives have overcome the disadvantages of the conventional MTA regarding the context of the study. Do these materials exhibit better sealing ability than MTA or do they just differ in composition? Is there any existing literature regarding the effect of different irrigants on the structural properties of MTA versus the proposed alternatives?

Our response: The composition and advantages of the newer MTA cements (Ortho-MTA and Retro-MTA) over the conventional ProRoot MTA cement has been mentioned in the revised manuscript. Also, the literature on the effect of different chelating agents on tricalcium based cements are added in the revised manuscript.

Question 3: With reference to the proposed alternatives, is there any contrasting evidence regarding the dislodgement resistance and structural changes after exposure to different irrigants or the idea is completely novel? If this is the case, please include this in the introduction section.

Our response: Till date no studies have evaluated the effect of maleic acid or Dual Rinse HEDP on the dislodgment resistance and structural changes of newer MTA cements which are used after its complete setting. This has been added now to the revised manuscript.

Question 4: Third paragraph in the introduction is very generic and can be improved within the context of the title of the study.

Our response: Third paragraph has been modified in the revised manuscript.

**Materials & Methods:**

Question 1: No clear description as to how the sample size was calculated for the current study; the quoted reference had a single group (Biodentine) and the only objective was to determine the pushout bond strength of Biodentine when exposed to different irrigation protocols. On the contrary, the present study had three groups, and the objective was twofold i.e., dislodgement resistance and structural changes of cements were evaluated. Hence, I believe that the study is somewhat underpowered.

Our response: Reference [20] was selected as a key reference for determining the sample size in the present study because of the similar nature of the primary outcome assessed. Based on this reference article, sample size was estimated to be 10 in each group at 95% CI and 80% power. The secondary objective of this study was more of a qualitative data than quantitative. Hence, experiments were performed in triplicates with the sample size of 3 in each group. This sentence has been added in the revised manuscript.

Question 2: Line 132: 0.2% sodium azide; is this a recommended storage medium?

Our response: Extracted teeth were stored in 0.9% saline containing 0.2% sodium azide prior to the experiments. This sentence has been added to the revised manuscript.

Question 3: Lines 192-193: why only fifteen specimens were evaluated for microstructure analysis? Is it because it was their secondary objective; if this is the case where did this number come from as this is not mentioned in the sample size calculation either?

Our response: In the structural analysis experiment since the data was of qualitative nature than quantitative, the experiments were performed in triplicates (n=3). Hence, only 15 specimens were evaluated.

**Results:**

Question 1: Was the data non-normal? I am assuming this was the case as non-parametric tests were applied.

Our response: Yes, the data was not normally distributed. Hence, non-parametric tests were used. This has been added now in the revised manuscript.

Question 2: Which statistical test was applied for the data in table 2?

Our response: Type of bond failures were assessed by Chi square test. This has been added in the revised manuscript.
